# A patient who experienced thyroid storm complicated by rhabdomyolysis, deep vein thrombosis, and a silent pulmonary embolism: a case report

**DOI:** 10.1186/1756-0500-6-198

**Published:** 2013-05-20

**Authors:** Taro Umezu, Keigo Ashitani, Takahiro Toda, Tatsuo Yanagawa

**Affiliations:** 1 Medical Intern, Nerima General Hospital, 1-24-1 Asahigaoka, Nerima-ku, Tokyo 176-8530, Japan; 2 Department of Internal Medicine, Nerima General Hospital, 1-24-1 Asahigaoka, Nerima-ku, Tokyo 176-8530, Japan

## Abstract

**Background:**

Thyroid storm is a serious condition of thyrotoxicosis. Hyperthyroidism often presents with thrombotic events, especially at cerebral sites; however, the possible association between a lower extremity deep vein thrombosis (LEDVT) and thyroid storm has not been previously reported. We encountered a patient who developed thyroid storm, associated with rhabdomyolysis, followed by LEDVT and a small silent pulmonary embolism (PE). The case is discussed with references to the pertinent literature.

**Case presentation:**

A 50-year-old woman with no past medical history was referred to our hospital because of severe diarrhea, muscle weakness in her lower limbs (manual muscle testing: MMT 3), and disturbances of consciousness. She was diagnosed as having Graves’ disease based on the presence of struma, exophthalmos, and hyperthyroidism with TSH receptor antibody positivity; we further determined that the patient was experiencing thyroid storm based on the results of the Burch-Wartofsky scoring system and a Japanese diagnostic criteria. Treatment with steroids, iodine potassium, methimazole, and propranolol was initiated. Severe watery diarrhea continued, and the laboratory data revealed hypokalemia (2.0 meq/L). On day 14, a blood analysis showed a sudden elevation in her creatinine kinase (CK) level, leading to a diagnosis of rhabdomyolysis. Thereafter, the muscle weakness in her lower limbs advanced to a degree of MMT 1. Seven days after the diagnosis of rhabdomyolysis, pitting edema began to appear in bilateral lower extremities. Contrast-enhanced CT scans revealed a LEDVT involving the left common iliac vein, bilateral femoral veins, and left popliteal vein. Furthermore, a small PE was identified. Hyperthyroidism often presents with thrombotic events, especially at cerebral sites, but few reports of PE or LEDVT have been made.

**Conclusion:**

This case suggests that the occurrence of thyroid storm may be associated with a risk of LEDVT and/or PE. We suggest that DVT preventive measures are undertaken, and that a lower limb venous echo or contrast-enhanced CT examination would be considered if LEDVT is suspected.

## Background

Thyroid storm is a serious condition of thyrotoxicosis that is diagnosed based on a clinical examination and is associated with a high mortality rate [1-3]. Hyperthyroidism often presents with thrombotic events, especially at cerebral sites [4,5]; however, few reports of lower extremity deep vein thrombosis (LEDVT) have been made. Recently, an increased risk of pulmonary embolism (PE) among patients with hyperthyroidism has been reported [6]. Most cases of PE arise from thrombi in the deep venous system of the lower extremities [7], suggesting that LEDVT may be more likely to occur among patients with hyperthyroidism. Furthermore, thyroid storm is likely associated with a much higher risk of LEDVT, because clinical conditions such as prolonged immobilization, congestive heart failure, dehydration, and the presence of a central venous catheter are common in patients experiencing thyroid storm and are known to predispose an individual to LEDVT. However, the possible association between LEDVT and thyroid storm has not been previously reported.

We encountered a patient who developed thyroid storm associated with rhabdomyolysis, followed by LEDVT and a small silent PE. The case is discussed with references to the pertinent literature.

## Case presentation

### Present illness

A 50-year-old Japanese woman with no past medical history was referred to our hospital. Two weeks prior to admission, she had developed fever, stomachache, and diarrhea. A diagnosis of gastroenteritis was made at a local clinic, and she was treated with antibiotics and intestinal medications; however, no improvement in her symptoms was seen. A week later, she began to experience lower extremity weakness, which soon led to a gait disturbance. As her consciousness began to deteriorate, she was transferred to and was examined at the Department of Emergency Medicine of our hospital; she was subsequently admitted.

### Personal history

Non-smoker, drinks alcohol occasionally.

### Past and family history

Not contributory.

### Condition at the time of admission

Glasgow coma scale score for consciousness, 10 (E3V2M5); blood pressure, 158/90 mmHg; pulse, 145 beats/min, irregular; body temperature, 38.3 C; respiratory rate, 30/min; oxygen saturation, 98% while she was breathing ambient air. A physical examination revealed struma, exophthalmos, and significant muscle weakness in the lower extremities; the strength in the quadriceps, hamstrings, tibialis anterior, and extensor halluces longus muscles was rated as 3 out of 5 using manual muscle testing (MMT). The patient’s reflexes were decreased. A chest radiography was clear, and the cardiothoracic ratio was 44%. An electrocardiogram revealed atrial fibrillation with a rapid ventricular response (pulse rate, 145/min). Echocardiography revealed no significant dilatation of the ventricles and normal wall motion with an ejection fraction of 59%.

### Blood examination findings on admission

Thyroid function tests showed severe thyrotoxicosis with a FreeT4 level > 6.0 ng/dL and a FreeT3 > 30.0 pg/mL, as well as a low TSH level of < 0.05 μU/mL with TSH receptor antibody positivity. She was diagnosed as having Graves’ disease. The serum concentration of brain natriuretic peptide was 109.2 pg/mL, which was suggestive of heart failure. An arterial blood gas analysis (in ambient air) revealed a pH of 7.530, a PCO_2_ of 31.2 torr, a PO_2_ of 81.0 torr, an HCO_3_ of 25.4 mEq/L, and a BE of 3.0, indicating respiratory alkalosis.

Based on the Burch-Wartofsky-score [1], which is a widely used global scale for evaluating the severity of thyrotoxicosis, the patient’s condition was diagnosed as thyroid storm (thyrotoxicosis with delirium [20 points], moderate heart failure [10 points], tachycardia of 145 beats/min [25 points], and atrial fibrillation [10 points]; a fever over 38.3C [15 points], diarrhea [10 points], for a total score of 90 points [61 points or higher is definitive of a thyroid storm]). The diagnosis was also supported by the Japanese diagnostic criteria [8] (Table 1).

**Table 1:** Blood examination findings on admission

**Variable**	**Unit**	**On admission**	**Reference range**
Free T3	(pg/ml)	>30.0	2.0–4.5
Free T4	(ng/dl)	>6.0	0.7–1.8
TSH	(μU/ml)	<0.05	0.3–4.5
TSH receptor antibody	(IU/liter)	>600	0–0.9
BNP	(pg/ml)	109.2	0–18.4
Arterial blood gas analysis (ambient air)			
pH		7.53	7.35–7.45
PaCO2	(mmHg)	31.2	35–45
PaO2	(mmHg)	81.0	80–100
Bicarbonate	(mmol/liter)	25.4	20–26
Base excess	(mmol/liter)	3.0	-3–3

### Clinical course

**Figure 1:**
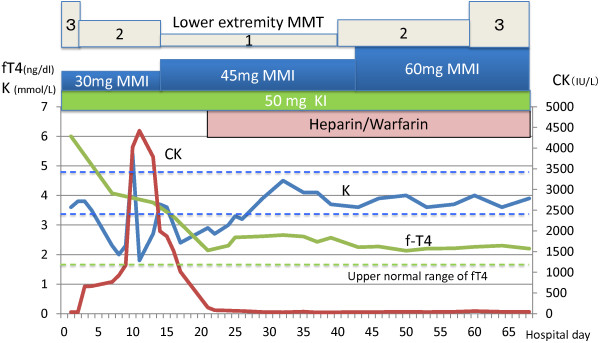
**Clinical course.** Serum fT4, CK (creatinine kinase), and K levels and the patient’s symptoms are shown, along with the administrations of MMI (methimazole), KI (potassium iodine), heparin, and warfarin. Muscle strength was rated from 1 to 5 according to MMT (manual muscle testing).

Treatment with steroids, 50 mg of iodine potassium (KI), 30 mg of methimazole (MMI), and propranolol (40 mg daily) was initiated. A central venous catheter was placed. Three days after admission, an electrocardiogram showed a “coronary T” wave. A coronary angiography was performed, and no evidence of coronary stenosis or cardiac dysfunction was seen. The severe watery diarrhea persisted, and the laboratory data revealed hypokalemia (2.0 meq/L). The control of the hyperthyroidism was difficult even with 60 mg of MMI until day 68. On day 14, a blood analysis showed a sudden elevation in creatinine kinase (CK) soaring to 4024 IU/L within a few days, with a CK-MM of 97%, leading to a diagnosis of rhabdomyolysis (Figure 1). The patient’s serum potassium concentrations were transiently elevated during the CK elevation, suggesting the release of CK from the skeletal muscle. Despite continuing antithyroid medications, fluids replacement, and electrolyte management, the muscle weakness in the hip joint and knees advanced to a degree of MMT 1, while the muscle strength in the upper limbs was rated as MMT 4.

**Figure 2:**
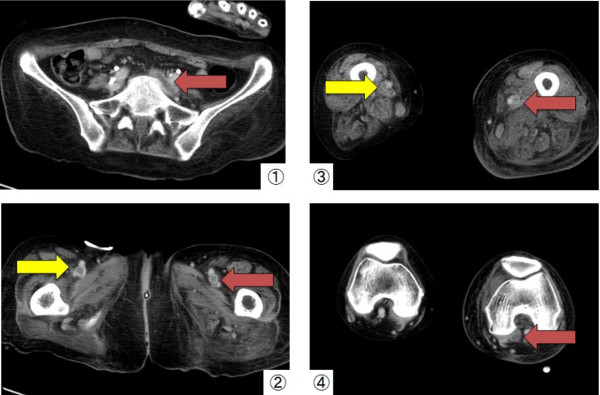
**Contrast-enhanced CT scans of the lower limbs.** Contrast-enhanced CT scans revealed a lower extremity deep vein thrombosis involving the left common iliac vein (**1**), bilateral femoral veins (**2**, **3**), and the left popliteal vein (**4**).

**Figure 3:**
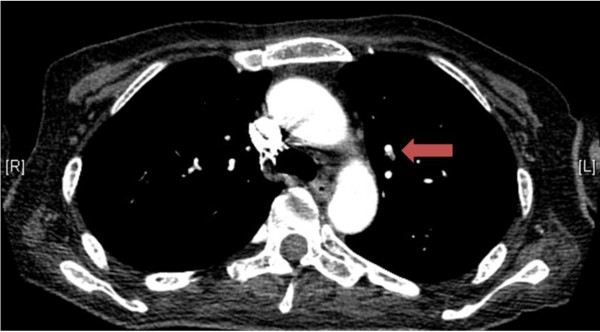
**Contrast-enhanced CT scans of the lung.** A small pulmonary embolism in the left upper lobe was identified.

Graduated compression stockings were used. Seven days after the diagnosis of rhabdomyolysis, pitting edema began to appear in bilateral lower extremities. Four days after the appearance of the pitting edema, the swelling worsened and tenderness developed in the left extremity. Contrast-enhanced CT scans revealed a LEDVT involving the left common iliac vein, bilateral femoral veins and left popliteal vein (Figure 2). Furthermore, a small PE in the left upper lobe was identified (Figure 3). The D-dimer level was 22.3 ng/dL. The coagulation factor VIII levels, which have been reported to be associated with thrombosis in Graves’ disease [9,10], were within the normal range. Treatment with heparin and warfarin was started. The LEDVT was minimized, and the PE was no longer identifiable on CT scans performed on day 90. Medical rehabilitation enabled her to recover her muscle strength to MMT 4. Once the thyroid function parameters had normalized, she was discharged from our hospital.

## Discussion

Thyroid storm is a serious condition of thyrotoxicosis that is diagnosed based on a clinical examination and is associated with a high mortality rate [1,2]. Our patient was diagnosed as having thyroid storm based on the Burch-Wartofsky-scoring system [1], and a Japanese diagnostic criteria [8]. The present case was complicated by rhabdomyolysis. Although hyperthyroid patients characteristically have normal or low serum levels of muscle enzymes, this case demonstrates that rhabdomyolysis can occur in patients with hyperthyroidism [11,12]. Circulatory collapse, hypokalemia and dehydration under the excessive hypermetabolic state presumably suppressed the supply of energy and oxygen to the muscle cells, leading to cellular damage. Of note, the LEDVT apparently occurred after the onset of rhabdomyolysis in the present case, possibly because of the immobilization caused by muscle damage. Increased factor VIII procoagulant protein may be a predisposing factor for DVT [10], but the factor VIII activity was not elevated in our patient. Hyperthyroidism is associated with an increased risk of venous thrombotic events. In particular, a number of case reports have documented acute venous thrombosis complications in patients with overt hyperthyroidism [4], especially at cerebral sites [5]. However the possible association between PE or LEDVT and hyperthyroidism has rarely been reported. Recently, Lin reported an increased risk of PE among patients with hyperthyroidism [6]. Most cases of PE arise from thrombi in the deep venous system of the lower extremities [7]. PE is a common complication of LEDVT, occurring in more than 50% of cases with phlebographically confirmed LEDVT [7]. One in every three patients with LEDVT may have a silent PE. This suggests that factors that promote the development of LEDVT also increase the risk for PE. In adults, the clinical conditions that predispose an individual to LEDVT are prolonged immobilization, congestive heart failure, dehydration, and the presence of a central venous catheter; these conditions are also common in patients experiencing thyroid storm. Furthermore, it has been reported that the risk of DVT to gradually rise with increasing levels of free thyroxine. In the absence of traditional acquired risk factors, free thyroxine levels above reference range, yielded a sex- and age-adjusted odds ratio of 13.0 [13]. However, many patients with LEDVT are often asymptomatic, and the association between LEDVT and thyroid storm may not have been reported.

## Conclusion

This case suggests that the occurrence of thyroid storm may be associated with a risk of LEDVT and/or PE. We suggest DVT preventive measures are undertaken, and that a lower limb venous echo or contrast-enhanced CT examination would be considered if LEDVT is suspected.

## Consent

“Written informed consent was obtained from the patient for publication of this case report and accompanying images. A copy of the written consent is available for review by the Editor-in-Chief of this journal.”

## Competing interests

The authors declare that they have no competing interests.

## Authors’ contributions

UT was the main contributor to the preparation of the rough draft. KA and TT were involved in the literature review and writing of the article. TY critically revised the manuscript. All authors read and approved the final manuscript.

## References

[B1] Burch HB Wartofsky L Life-threatening thyrotoxicosis: thyroid storm Endocrinol Metab Clin North Am 1993 22 263 277 8325286

[B2] Chong HW See KC Phua J Thyroid storm with multiorgan failure Thyroid 2010 20 333 336 10.1089/thy.2009.0181 20146655

[B3] Yoshino T Kawano D Azuhata T Kuwana T Kogawa R Sakurai A Tanjoh K Yanagawa T A patient with Graves’ disease who survived despite developing thyroid storm and lactic acidosis Ups J Med Sci 2010 115 282 286 10.3109/03009734.2010.486908 20731531 PMC2971487

[B4] Franchini M Lippi G Targher G Hyperthyroidism and venous thrombosis: a casual or causal association? A systematic literature review Clin Appl Thromb Hemost 2011 17 387 392 10.1177/1076029610364521 20308227

[B5] Verberne HJ Fliers E Prummel MF Stam J Brandjes DP Wiersinga WM Thyrotoxicosis as a predisposing factor for cerebral venous thrombosis Thyroid 2000 10 607 661 10.1089/thy.2000.10.607 10958314

[B6] Lin HC Yang LY Kang JH Increased risk of pulmonary embolism among patients with hyperthyroidism: a 5-year follow-up study J Thromb Haemost 2010 8 2176 2181 10.1111/j.1538-7836.2010.03993.x 20738759

[B7] Tzoran I Saharov G Brenner B Delsart D Román P Visoná A Jiménez D Monreal M The RIETE Investigators Silent pulmonary embolism in patients with proximal deep vein thrombosis in the lower limbs J Thromb Haemost 2012 10 564 571 10.1111/j.1538-7836.2012.04648.x 22288520

[B8] Akamizu T Satoh T Isozaki O Suzuki A Wakino S Iburi T Tsuboi K Monden T Kouki T Otani H Teramukai S Uehara R Nakamura Y Nagai M Mori M Diagnostic criteria and clinical features, and incidence of thyroid storm based on nationwide surveys Thyroid 2012 22 661 679 10.1089/thy.2011.0334 22690898 PMC3387770

[B9] Koster T Blann AD Briët E Vandenbroucke JP Rosendaal FR Role of clotting factor VIII in effect of von Willebrand factor on occurrence of deep-vein thrombosis Lancet 1995 345 152 155 10.1016/S0140-6736(95)90166-3 7823669

[B10] Maes J Michotte A Velkeniers B Stadnik T Jochmans K Hyperthyroidism with increased factor VIII procoagulant protein as a predisposing factor for cerebral venous thrombosis J Neurol Neurosurg Psychiatry 2002 73 458 10.1136/jnnp.73.4.458 12235324 PMC1738073

[B11] Bennett WR Huston DP Rhabdomyolysis in thyroid storm Am J Med 1984 77 733 735 10.1016/0002-9343(84)90375-9 6486150

[B12] Hosojima H Iwasaki R Miyauchi E Okada H Morimoto S Rhabdomyolysis accompanying thyroid crisis: an autopsy case report Intern Med 1992 31 1233 1235 10.2169/internalmedicine.31.1233 1286234

[B13] van Zaane B Squizzato A Huijgen R van Zanten AP Fliers E Cannegieter SC Büller HR Gerdes VE Brandjes DP Increasing levels of free thyroxine as a risk factor for a first venous thrombosis: a case–control study Blood 2010 115 4344 4349 10.1182/blood-2009-11-253724 20308594 PMC2881489

